# EXPLORING RACIAL-ETHNICITY DIFFERENCES IN SERVICES USED BY GRANDPARENTS RAISING GRANDCHILDREN

**DOI:** 10.1093/geroni/igz038.2492

**Published:** 2019-11-08

**Authors:** Tamar E Shovali, Kerstin G Emerson

**Affiliations:** 1 Eckerd College, St. Petersburg, Florida, United States; 2 University of Georgia, Athens, Georgia, United States

## Abstract

Nearly three million grandparents in the US serve as primary caregivers for their grandchildren. Little research on formal service use and grandfamilies exists for Black and Hispanic populations. To begin to address this gap we conducted exploratory analyses using nationally representative estimates of characteristics and service accessibility of noninstitutionalized children living with grandparents from the 2013 National Survey of Children in Nonparental Care. Our goal was to understand differences in service use as a function of grandfamily race/ethnicity. We specifically explored grandparents’ formal service count, financial services received, confidence in obtaining/using community services, and level of role preparation by race/ethnicity. We calculated descriptive statistics for these service variables for grandparents raising Hispanic, White, Black, and Other identified grandchildren (N = 892). On average, there was a minimal range for the number of formal services used (M range = 5.26 – 5.84, possible = 0 – 10 higher equals more services used), reported number of financial services (M range = 0.71 - 0.78, possible = 0 – 3 higher equals more financial services received), and confidence obtaining/using services (M range = 7.4—7.9, possible = 1 – 9 higher equal more confidence). Most prepared to take on the caregiving role were grandparents of White children (55%) followed by Black (21.6%), Other (12.3%), and Hispanic (11.1%) indicating that although grandparents in this sample report being confident and able to access formal services, grandparents of White children report being feeling more prepared to take on caregiving than grandparents of Black, Hispanic, and Other combined.

